# *Hospitalitermes krishnai*, a new nasute termite (Nasutitermitinae, Termitidae, Isoptera), from southern Sumatra, Indonesia

**DOI:** 10.3897/zookeys.148.1768

**Published:** 2011-11-21

**Authors:** Syaukani, Graham J. Thompson, Seiki Yamane

**Affiliations:** 1 Department of Biology, Faculty of Mathematics and Natural Sciences, Syiah Kuala University, Darussalam 23111, Banda Aceh, Indonesia; 2 Department of Biology, Faculty of Science, University of Western Ontario, 1151 Richmond Road North, London N6A 5B7, Ontario, Canada; 3 Department of Earth and Environmental Science, Graduate School of Science and Engineering, Kagoshima University, Kagoshima 890-0065, Japan

**Keywords:** Nasutitermitinae, *Hospitalitermes*, nasute termite, new species, Sumatra

## Abstract

A new species of nasute termite, *Hospitalitermes krishnai* **sp. n.**, is described from soldiers and workers discovered in Lampung Province, Sumatra. This species can be distinguished from other related *Hospitalitermes* species from Southeast Asia by the anterior part of head capsule that is much smaller than the posterior part, head capsule that is moderately constricted behind the antennal sockets, and relatively deep depression between the head and nasus and, finally, the short and robust nasus measuring less than half as long as head capsule. Moreover, in profile the nasus is slightly up-curved but slightly decurved at the apical tip. We name this new species after Professor Kumar Krishna in recognition of his life-long contributions to termite taxonomy, systematics and biology.

## Introduction

*Hospitalitermes* Holmgrenis one of only a few termite genera, together with *Lacessititermes* and *Longipeditermes*, that forage in open-air processional columns (Tho 1992, Jones and Gathorne-Hardy 1995, Miura and Matsumoto 1998). These three genera are phylogenetically very closely related (Inward et al. 2007). *Hospitalitermes* has long been treated as a distinct genus within the nasute termites (Holmgren 1913), but morphological characters distinguishing *Hospitalitermes* from related genera are subtle (e.g. Tho 1992, Gathorne-Hardy 2001, Syaukani^ 2010^). *Hospitalitermes bicolor* (Haviland), *Hospitalitermes ferrugineus* (John), *Hospitalitermes flaviventris* (Wasmann), *Hospitalitermes flavoantennaris* Oshima, *Hospitalitermes grassii* Ghidini, *Hospitalitermes hospitalis* (Haviland), *Hospitalitermes medioflavus* (Holmgren), *Hospitalitermes umbrinus* (Holmgren) and *Hospitalitermes seikii* Syaukani have all been collected from the island of Sumatra in Indonesia. In all of these species, the workers typically forage in the afternoon and evening *en masse*. They are conspicuous in the forests of Southeast Asia where their foraging parties can approach half a million individuals (Collins 1979). These foraging parties are composed of a minority of defensive nasute soldiers that protect a majority of workers. In this paper we describe *Hospitalitermes krishnai* sp. n. based on a series of specimens collected from southern Sumatra, Indonesia.

## Material and Methods

Specimens of *Hospitalitermes krishnai* sp. n. were collected from a processional column on the forest floor at Sumber Jaya, Kotabumi, Lampung Province, Sumatra on 18^th^ September 2007. We photographed the head, body (in profile) and pronotum of the soldier caste (preserved in 80% ethanol) using a digital microscope (HFVH-8000, Keyence, Osaka). Further, we removed mandibles of the worker caste for closer examinations. We then examined these specimens for diagnostic characters on glass slides mounted with Euparal 3C 239 (Waldeck GmbH & Co. KG, Muenster). We photographed the specimens using a conventional digital camera (Coolpix 3340, Nikon, Tokyo) attached to a Nikon Eclipse E600 lense. From these images, we constructed multi-focused montages using Helicon Focus 4.03 Pro software (Helicon Soft Ltd, Kharkov). General morphological terminology used for describing soldiers and workers follows those of Tho (1992), Sands (1998) and Gathorne-Hardy (2001).

### Mesurements

Measurements of the soldier body parts follow those in Roonwal and Chhotani (1989) and Tho (1992). Measurements were made for the soldier caste as follows: head length including nasus (HLN), head length to base of mandibles (HL), nasus length (NL), head width at point of constriction (HWC), maximum head width (HW), maximum height of head excluding postmentum (HH), and length (PL) and width (PW) of pronotum. We also calculated the ratio of NL to HL.

## Systematics

**Family Termitidae Latreille, 1802**

**Genus *Hospitalitermes* Holmgren, 1913**

### Hospitalitermes krishnai sp. n.

urn:lsid:zoobank.org:act:E6228361-6581-46F2-930D-863E7B438010

http://species-id.net/wiki/Hospitalitermes_krishnai

Figs 1 [Fig F2] 7

#### Description.

**Alates**. Not available

#### Soldier.

**Figures 1–3.**
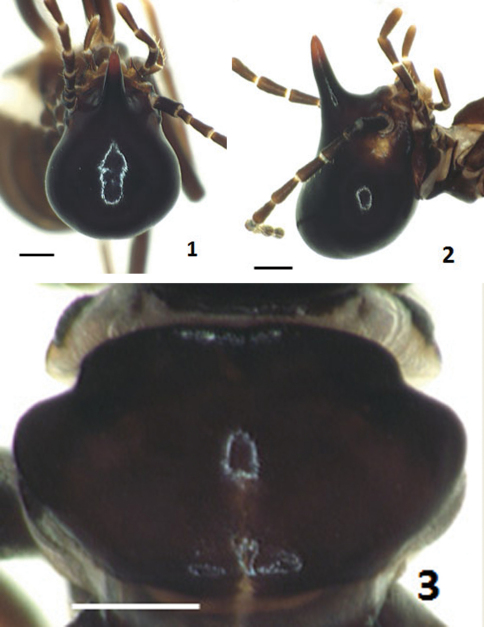
Soldiers of *Hospitalitermes krishnai* sp. n. Head in dorsal view **1**, head in profile **2**, and pronotum **3**. Scale bar: 0.3 mm **1, 2**, 0.2 mm **3**.

**Figures 4–6. F2:**
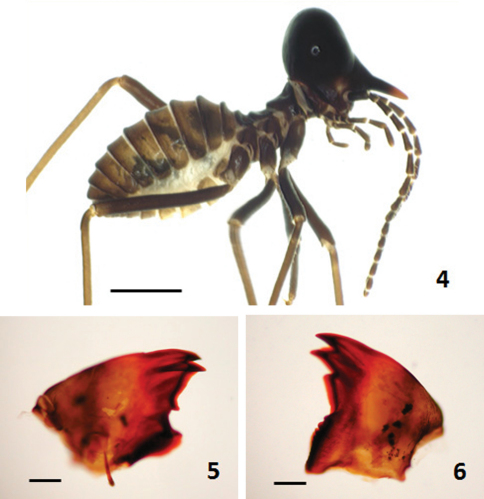
*Hospitalitermes krishnai* sp. n. Soldier **4** and workers **5–6**. Habitus in profile **4**, left **5** and right **6** mandibles. Scale bar: 0.5 mm **4**, 0.1 mm **5–6**.

(Figs 1–4). Monomorphic. Head capsule entirely black (with indistinct spots behind antennal sockets); nasus with apical third lighter and basal two-thirds darker; antenna (except for the first segment) uniformly sepia brown to dark sepia brown, paler than head capsule. Pronotum in dorsal view slightly paler than or similar to head capsule in coloration. Abdominal tergites dark brown to blackish brown. Coxae and femora sepia brown to dark sepia brown; tibiae pale brown to brown. Head capsule in dorsal view moderately constricted behind antennal sockets, with anterior part excluding nasus extremely smaller than posterior part in size; median portion of its posterior margin nearly straight; dorsal outline (including nasus) in profile moderately concave (i.e., showing a depression). Nasus in dorsal view relatively short and robust, less than half as long as head capsule, in profile slightly up-curved but apical third feebly down-curved. Antenna with 14 segments; third segment longer than fourth; fourth and fifth nearly equal in length, the former slightly broader than the later; 6^th^-14^th^ gradually decreasing in length. Pronotum in dorsal view with anterior margin very feebly indented in the middle and posterior margin roundly convex.

#### Worker.

(Figs 5–6) Dimorphic. Head capsule dark brown to black. Epicranial suture brown. Fontanel brown to dark brown. Labrum yellowish to brown. Clypeus brown to blackish brown. Anticlypeus yellowish. Antennasepia brown except for the first segment. Antenna consisting of 15 segments; third segment longer than fourth; fourth slightly shorter than or equal to fifth; 6^th^-15^th^ gradually increasing in length. **Left mandible:** apical tooth clearly shorter than first marginal tooth; anterior edge of first marginal tooth distinctly longer than posterior edge; second marginal tooth absent, third marginal tooth smaller than first marginal tooth, but fairly protruding from cutting edge and separated from molar prominence by a distinct gap; fourth marginal tooth retracted, completely hiding behind molar prominence. **Right mandible:** first marginal tooth with anterior edge almost straight; second marginal tooth clearly recognized and separated from much larger first marginal tooth; posterior edge of second marginal tooth nearly straight; outline of molar plate slightly visible; cockroach notch of molar plate absent.

**Table 1.** Measurements (in mm) for 20 soldiers of *Hospitalitermes krishnai* sp. n.

**Character**	**Holotype**	**Range**
Head length including nasus (HLN)	1.95	1.75-1.95
Head length measured to base of mandible (HL)	1.51	1.45-1.51
Nasus length (NL)	0.51	0.44-0.51
Nasus index = NL/HL	0.33	0.30-0.33
Head width at point of constriction (HWC)	0.85	0.78-0.86
Maximum head width (HW)	1.22	1.15-1.22
Maximum height of head excluding postmentum (HH)	0.95	0.82-0.95
Pronotum length (PL)	0.47	0.41-0.47
Pronotum width (PW)	0.80	0.75-0.80

Note: the holotype has the largest value in range for nearly all characters.

#### Comparisons.

In the soldier caste, *Hospitalitermes krishnai* sp. n. differs from *Hospitalitermes birmanicus* Snyder both in the shape of the head capsule and nasus in dorsal view. The coloration of both antennae and tibiae (pale brown to dark sepia brown) distinguishes *Hospitalitermes krishnai* sp. n. from *Hospitalitermes umbrinus* (Haviland) and *Hospitalitermes diurnus* Kemner. In *Hospitalitermes krishnai* the nasus is less than half as long as the head capsule; this distinguishes it from *Hospitalitermes hospitalis* (Haviland), *Hospitalitermes medioflavus* (Holmgren), and *Hospitalitermes lividiceps* (Holmgren) in which the nasus is more than half as long as head capsule. Finally, *Hospitalitermes krishnai* is distinguished from *Hospitalitermes seikii* Syaukani by the gold-orange abdominal tergites in the latter species.

This species can be distinguished from other related *Hospitalitermes* from Southeast Asia bythe anterior part of the head capsule that is much smaller than the posterior part, the head capsule that is constricted behind the antennal sockets, and the relatively deep depression between the head and nasus and, finally, the short and robust nasus measuring less than half as long as head capsule.

From the examination of thousands of specimens of *Hospitalitermes* from the Syaukani personal collection, as well as a number of type series at the Natural History Museum (London), we note that the pilosity cannot be used as a reliable character for identification since specimens from different *Hospitalitermes* colonies appear extremely variable in this character. We therefore do not consider pilosity here. Moreover, we think that similar variation in the concavity of the head capsule may occur in some related species (Chhotani 1997), and that soldier “eyes” described by Chhotani are actually just indistinct spots. Strictly, speaking *Hospitalitermes* soldiers do not have eyes.

**Figure 7.**
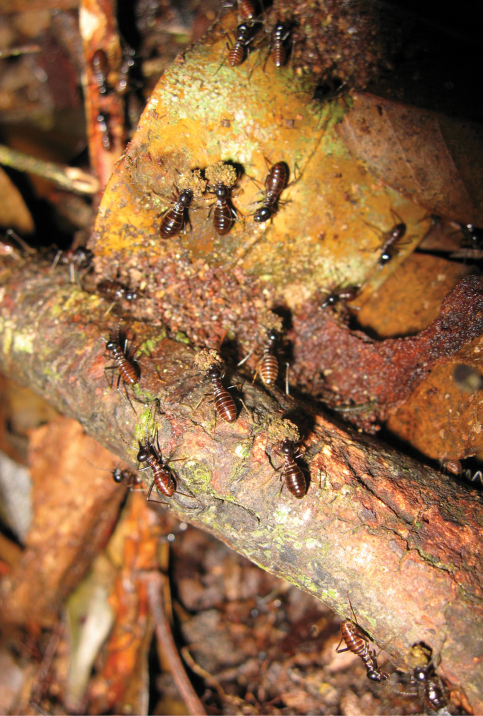
Soldiers and workers of *Hospitalitermes krishnai* sp. n are in processional column on forest floor. Workers are carrying food-balls and returning to the nest. Photo taken by Syaukani (2007).

#### Material Examined.

Holotype:soldier collected in the afternoon from a mass processional column on the forest floor (very steep slope) in an undisturbed lowland/sub-montane rain forest (1.250 m in altitude), Sumber Jaya (4°47'16"S, 103°35'8"E), Kotabumi, Lampung Province, Sumatra. The nest was not located. Syaukani leg., 18 September, 2007. Colony code: SY-2007-LP-0092. The holotype is deposited at Museum Zoologicum Bogoriense, Cibinong, Indonesia. Paratypes (soldiers and workers from the same colony from which the holotype was collected) are deposited at Museum Zoologicum Bogoriense, Cibinong (Indonesia), the Natural History Museum, London (UK), Syiah Kuala University, Darussalam, Banda Aceh (Indonesia), the Kitakyushu Museum of Natural History and Human History (Japan), and the American Museum of Natural History, New York (USA).

#### Etymology.

This species is named after Professor Kumar Krishna who has made significant, life-long contributions to the knowledge of the taxonomy, systematics and biology of termites.

## Discussion

This study contributes to the knowledge of termite diversity in Sumatra, describing one new species of termite with above-ground processional foraging. From morphology it is difficult to separate the genus *Hospitalitermes* from the related *Lacessititermes* (also with processional foraging), but the presence of a notch on the molar plate of the worker right mandible in *Lacessititermes* is sufficient to distinguish the former from the later (Tho 1992, Gathorne-Hardy 2001, Syaukani^ 2008^, 10) (*Hospitalitermes* lacks this notch). Our description of *Hospitalitermes krishnai* relies on colour, and while this character can be problematic for identification - for example, if color fades over the time - we also find that even type series stored for over a hundred years (e.g. *Hospitalitermes bicolor* that collected by Haviland in 1894), the color has remained adequate and suitable for recognizing the species. In our experience, a combination of colour and other morphological characters are important when identifying *Hospitalitermes* species. Indistinct spots behind antennal sockets of some species are not eyes. Soldiers of *Hospitalitermes* have no eyes.

*Hospitalitermes krishnai* sp. n. is notable because of its peculiar above-ground foraging. Furthermore it shows a distinct size dimorphism with large and small workers. This phenomenon, though rare among termites, has been previously noted for related species. For example, Tho (1992) reported a dimorphic worker caste in *Hospitalitermes hospitalis* from Peninsular Malaysia. Likewise Miura (2004) distinguished three types of worker (major, medium and small) in *Hospitalitermes medioflavus* based on material collected from Borneo. These examples may be interesting to understand the division of labour among workers in a colony. It is the evolution of morphologically distinct worker castes that is famously referred to as Darwin’s “special difficulty” (Ratnieks et al. 2011).

We suspect there remain many undescribed species of *Hospitalitermes* in Sumatra. The diversity of previously undescribed termites in this region may stem from the island isolation and resulting high degree of animal endemism.

## Supplementary Material

XML Treatment for Hospitalitermes krishnai
